# Huang Qi Jian Zhong Pellet Attenuates TNBS-Induced Colitis in Rats via Mechanisms Involving Improvement of Energy Metabolism

**DOI:** 10.1155/2013/574629

**Published:** 2013-06-06

**Authors:** Duan-Yong Liu, Chun-Shui Pan, Yu-Ying Liu, Xiao-Hong Wei, Chang-Man Zhou, Kai Sun, Ke He, Chong Li, Li Yan, Jing-Yu Fan, Chuan-She Wang, Toshifumi Hibi, Hong-Ning Liu, Jing-Yan Han

**Affiliations:** ^1^Jiangxi University of Traditional Chinese Medicine, Nanchang, Jiangxi 330004, China; ^2^Tasly Microcirculation Research Center, Peking University Health Science Center, Beijing 100191, China; ^3^Department of Integration of Chinese and Western Medicine, School of Basic Medical Sciences, Peking University, 38 Xueyuan Road, Beijing 100191, China; ^4^Department of Anatomy, School of Basic Medical Sciences, Peking University, Beijing 100191, China; ^5^Key Laboratory of Microcirculation, State Administration of Traditional Chinese Medicine, Beijing 100191, China; ^6^Department of Internal Medicine, Keio University School of Medicine, Tokyo 160-8582, Japan; ^7^Key Laboratory of Modern Preparation of TCM, Ministry of Education, Jiangxi University of TCM, 18 Yunwan Road, Nanchang, Jiangxi 330004, China

## Abstract

Huang Qi Jian Zhong Pellet (HQJZ) is a famous Chinese medicine formula for treatment of various gastrointestinal tract diseases. This study investigated the role of HQJZ in 2,4,6-trinitrobenzene sulfonic acid- (TNBS-) induced colitis and its underlying mechanism. Colonic mucosal injury was induced by TNBS in the Sprague-Dawley rats. In the HQJZ treatment group, HQJZ was administered (2 g/kg) for 14 days starting from day 1 after TNBS infusion. Colonic mucosal injury occurred obviously 1 day after TNBS challenge and did not recover distinctively until day 15, including an increase in macro- and microscopic scores, a colonic weight index, a decrease in colonic length, a number of functional capillaries, and blood flow. Inverted intravital microscopy and ELISA showed colonic microcirculatory disturbances and inflammatory responses after TNBS stimulation, respectively. TNBS decreased occludin, RhoA, and ROCK-I, while increasing Rac-1, PAK-1, and phosphorylated myosin light chain. In addition, ATP content and ATP5D expression in colonic mucosa decreased after TNBS challenge. Impressively, treatment with HQJZ significantly attenuated all of the alterations evoked by TNBS, promoting the recovery of colonic injury. The present study demonstrated HQJZ as a multitargeting management for colonic mucosal injury, which set in motion mechanisms involving improvement of energy metabolism.

## 1. Introduction

Inflammatory bowel disease (IBD) is chronic and relapsing inflammatory conditions, characterized by mucosal ulceration [1–3]. Previous studies indicated that the destroyed integrality of colonic epithelium and disturbances of colonic microcirculation occur in the colonic mucosal injury [4–6]. IBD is thought to be caused by impaired innate immunity. Treatment with anti-inflammatory drugs, immunosuppression, and biological therapy targeting specific components of the immune response is thus currently used, in addition to surgery, in clinic for the patients with IBD. However, the efficiency of these strategies remains unsatisfying, appealing to development of novel management. 

 Energy status is a fundamental regulator of cellular function, and its deficit has been considered to be a pathogenic factor in various conditions including IBD in human. Malnutrition and energy expenditure in IBD lead to energy deficit (ATP depletion) in colonic mucosa, resulting in the restitution of subnormal epithelial cell with hyper-permeability, edema, and the infiltration of inflammatory cell [7–10]. In experimental colitis, the concentration of adenine nucleotides is decreased in the colon, while administration of the adenine nucleotides ADP and ATP promotes epithelial cell restitution in damaged tissues [11, 12]. In addition, AMP-activated protein kinase (AMPK) was reported being down-regulated in the inflammatory colonic mucosa [13, 14]. AMPK is an energy-sensing enzyme. A recently published study revealed that AMPK activity supports endothelial barrier function by activating Rac/Cdc42/PAK pathway [15], which are known to play a critical role in endothelial barrier function via regulating cell adhesion and cytoskeleton dynamics. Activation of RhoA has been reported not only to evoke phosphorylation and degradation of occludin [16], but also to promote phosphorylation of myosin light chain (p-MLC), which interacts with actin generating cell contraction and leading to an impairment of barrier function [17–21]. Collectively, current evidence suggests that manipulating energy metabolism either by increasing ATP availability or by activating AMPK may be a potential management for IBD.

Huang Qi Jian Zhong Pellet (HQJZ) is composed of *Astragalus*, *Ramulus cinnamomi*, *White Peony root*, *Zingiber officinale *Roscoe, *Fructus jujubae*, *Radix glycyrrhizae*, and *Saccharum granorum* (Table 1). As a famous Chinese medicine formula, it has been used to treat various gastrointestinal tract diseases, such as gastritis and stomach ulcer. However, the mechanism responsible for its beneficial role is poorly understood. On the other hand, increased study has been published to explore the pharmacology of the composed herbs of HQJZ, showing the potential of this formula in anti-inflammation [22–24], antioxidative stress [25–27], and endothelial and mucosal protection [28, 29]. These results support the application of HQJZ in IBD [30]. Furthermore, study showed that *Astragalus* (one of main components of HQJZ) extract increases the levels of ATP and ADP and the activity of Na(+)-K(+)-ATPase, improves energy metabolism, and inhibits apoptosis, alleviating neuron injury after cerebral ischemia [31]. We speculated that HQJZ may be beneficial for IBD by acting at multiple targets involving regulation of energy metabolism. The present study was to address the role of HQJZ in 2,4,6-trinitrobenzene sulfonic acid- (TNBS-) induced colitis in rats and its underlying mechanism.

## 2. Materials and Methods

### 2.1. Animals

Male Sprague-Dawley rats weighing 180 to 220 g were purchased from the Animal Center of Peking University Health Science Center (The animal certificate number was SCXK 2006-0008). All animals were caged at 22 ± 2°C with a humidity of 50% ± 5% in a 12 h light/dark cycle and were provided standard diet and water *ad libitum*. The animals were fasted for 12 h before experiment but were allowed free access to water, and handled according to the guidelines of the Peking University Animal Research Committee. The surgical procedures and experimental protocols were approved by Peking University Biomedical Ethics Committee Experimental Animal Ethics Branch (LA2011-66).

### 2.2. Drugs

HQJZ (Batch no. 110310) was produced by TianQi pharmaceutical company (ChiFeng, China). The processing of the product followed strict quality control, and the ingredients were subjected to standardization. TNBS was purchased from Sigma (St. Louis, MO, USA). 

### 2.3. Colonic Mucosal Injury

Colonic mucosal injury was induced by TNBS in rats as reported previously [33]. Rat was anesthetized with pentobarbital (60 mg/kg, i.p.) and was administrated with TNBS through enema at a dose of 100 mg/kg. For this purpose, a 3% TNBS solution (w/v) was prepared by mixing 5% TNBS water solution with 30% ethanol at 4 : 3, and the freshly prepared solution was rectally instilled into the colon 8 cm proximal to the anus, in a volume depending on the rat, by a polyvinyl rubber catheter 2 mm in diameter. The rat was maintained in a head-down position for 15 min.

### 2.4. Experimental Protocol

Total seventy rats were randomly divided into 5 groups, 16 rats each: Sham 15 d group (the rats received physiological saline by enema and 24 h thereafter physiological saline by gavage for 14 days), Sham 15 d + HQJZ group (the rats received physiological saline by enema and 24 h thereafter HQJZ at 2 g/kg by gavage for 14 days), TNBS 1 d group (the rats received TNBS by enema and were sacrificed after 24 h), TNBS 15 d group (the rats received TNBS by enema and 24 h thereafter physiological saline by gavage for 14 days), and TNBS 15 d + HQJZ group (the rats received TNBS by enema and 24 h thereafter HQJZ at 2 g/kg everyday by gavage for 14 days). On day 15, all animals were sacrificed after anesthesia with urethane. The number of animals for assessment of various parameters in each group is detailed in Table 2.

### 2.5. Measurement of Colonic Blood Flow

Colonic blood flow (*n* = 8 for each group) was measured by a Laser Doppler perfusion image system (PeriScan PIM3 System; PERIMED, Stockholm, Sweden). On day 15, an incision was made through abdominal wall to expose peritoneal cavity under anesthesia with intraperitoneally administrated urethane (2.0 g/kg). Epicolic tissues were covered with black soft leather, and a segment of 1 to 5 cm colon above the anus was exposed. The scanning procedures were performed according to the studies of Paris et al. [34] and Huang et al. [35].

### 2.6. Macroscopical Evaluation

Colon was removed immediately from animal after being sacrificed, measured for its length, and opened longitudinally along colonic mesentery to clear its contents. Colon weight index (colonic weight/body weight × 100%) was calculated (*n* = 8). The scoring of colonic macroscopic damage (*n* = 8) was undertaken as described by Butzner et al. [36]. The criteria for assessment of macroscopic colonic damage were as follows: 0 score: normal appearance; 1 score: focal hyperaemia, no ulcers; 2 scores: ulceration without hyperaemia or bowel wall thickening; 3 scores: ulceration with inflammation at one site; 4 scores: ≥two sites of ulceration and inflammation; 5 scores: major sites of damage extending >1 cm along the length of the colon; and 6–10 scores: damage extended to >2 cm along the length of the colon, increasing the score by one for each additional cm of damage.

### 2.7. Microscopical Evaluation

The specimens were processed for paraffin sectioning and hematoxylin-eosin (HE) staining (*n* = 8). The microscopical evaluation was undertaken as described [37], taking into consideration both inflammatory cell infiltration and tissue damage. Scores for infiltration are as follows: 0: no infiltration; 1: increased number of inflammatory cells in the lamina propria; 2: inflammatory cells extending into the submucosa; and 3: transmural inflammatory cell infiltration. The scores for tissue damage are as follows: 0: no mucosal damage; 1: discrete epithelial lesions; 2: erosions or focal ulcerations; and 3: severe mucosal damage with extensive ulceration extending into the bowel wall.

### 2.8. Microcirculation in Colonic Chorion and Mucous Layer

Colonic microcirculation (*n* = 6 for each group) was observed under an inverted intravital microscope as described [38, 39]. The rats were anesthetized with urethane (2.0 g/kg body weight, intramuscularly). FITC (50 mg/kg, Sigma-Aldrich, St. Louis, MO, USA), rhodamine 6 G (0.1 mg/kg, Sigma-Aldrich, St. Louis, MO, USA), or physiological saline was infused via right internal jugular vein. Following a median laparotomy, the colon segment was exteriorized and placed on a special stage. The colon was antimesentericly opened to assess functional capillary density, leukocyte rolling and adhering, and albumin leakage in chorion and mucous layer. The animals were placed on thermostat-controlled heating pads to keep the body temperature at 37°C and protect from drying by warm physiological saline. Fluorescent images were acquired by an inverted fluorescence microscope (DM-LFS, Leica, Mannheim, Germany) 3 min after infusion of FITC-albumin and rhodamine 6 G. Leukocyte adhering and rolling and albumin leakage were evaluated as described previously [35, 39, 40]. 

### 2.9. Expression of CD11b on Neutrophils and Concentration of TNF-*α* in Colonic Tissue

Blood (*n* = 6) was collected and anticoagulated with heparin. Fifty microliters of blood was incubated with 0.5 *μ*g FITC-conjugated anti-CD11b antibody (BD Biosciences, San Jose, CA, USA) for 20 min. The mean fluorescence intensity of CD11b was accessed with a flow cytometer (FACS Calibur, BD Biosciences, San Jose, CA, USA). Neutrophils were sorted by characteristic forward/side-scatter expression. Five thousand neutrophils were evaluated for each sample [41]. 

Colonic tissue homogenate (*n* = 6) was prepared for measuring level of cytokines and Western blot analysis [42]. Level of TNF-*α* in colonic tissue homogenate was assessed by flow cytometry (FACS Calibur, BD Biosciences, San Jose, CA, USA) with a BD cytometric bead array kit (BD Biosciences Pharmingen, San Jose, CA, USA). The data were analyzed by the BD cytometric bead array analysis software [43].

### 2.10. Immunohistochemistry Staining of MPO, F-Actin, and Occludin in Colon

The tissue sections (*n* = 3) were treated with 0.3% H_2_O_2_ in methanol for 15 min and blocked by 3% normal goat serum. MPO immunohistochemistry was conducted as routine using a rabbit anti-MPO antibody (1 : 200, Santa Cruz, CA, USA). The images were captured by a digital camera connected to a microscope (BX512DP70, Olympus, Tokyo, Japan) [39]. For observation of F-actin and occludin, immunofluorescence staining and confocal microscopy (*n* = 3 for each group) were performed as described [35]. The sections were treated with 0.01 M sodium citrate for antigen retrieval, blocked with 3% normal goat serum at room temperature for 15 min, and then incubated with rabbit anti-occludin antibody (1 : 80, Abcam, Cambridge, UK) overnight at 4°C. After washing, colonic tissues were incubated with a secondary antibody, Dylight 488-labeled goat anti-rabbit IgG (KPL, Gaithersburg, MD, USA) for 2 h at 37°C in the dark. Hoechst 33342 (BD Biosciences Pharmingen, San Jose, CA, USA) was applied to stain nucleus. F-actin in colonic tissues was stained with phalloidine (1 : 40, Abcam, Cambridge, UK). All sections were photographed under a laser scanning confocal microscope (TCS SP5, Leica, Mannheim, Germany).

### 2.11. Enzyme-Linked Immunosorbent Assay

Enzyme-linked immunosorbent assay (ELISA) (*n* = 8) was performed according to the manufacturers' instruction (GBD, San Diego, CA, USA). Colonic tissues were lysed in RIPA buffer (50 mM Tris-HCl at pH 7.4, 150 mM sodium chloride, 1% NP-40, 0.5% sodium deoxycholate, and 0.1% sodium dodecyl sulfate) with protease and phosphate inhibitor cocktail (Merk, Ashland, MA, USA) using a sonicator. Crude lysates were centrifuged at 19357 g for 20 min. The supernatant was used to measure the level of ATP, ADP, and AMP (GBD, San Diego, CA, USA), as well as MPO, IL-10, and IL-6 (GBD, San Diego, CA, USA). Absorbance was read at 450 nm.

### 2.12. Western Blot Analysis

Western blot analysis (*n* = 5 for each group) was preformed as described previously [35]. Briefly, proteins were separated using a 10% Tri-HCL precast gel, and polyacrylamide electrophoresis (Bio-Rad Laboratories, Hercules, CA, USA) was conducted at 80 V for 90 to 120 min, and then the proteins were transferred to polyvinylidene fluoride membranes with 220 mA at 4°C for 2 h. The membranes were blocked with 5% nonfat milk or 5% BSA diluted in TBST for 1 h at room temperature and were incubated overnight with primary antibodies. The primary antibodies used were as follows: rabbit anti-GAPDH (1 : 2000), RhoA (1 : 2000), ROCK-I (1 : 2000), AMPK-*α* (1 : 1000), phospho-AMPK-*α* (1 : 800), PAK-1 (1 : 1000), phospho-MLC2 (1 : 800), occludin (1 : 500), and mouse anti-Rac-1 (1 : 1000), which were all from Abcam, Cambridge, UK, as well as goat anti-ATP5D (1 : 200) (Santa Cruz, CA, USA). The membrane was incubated with horseradish peroxidase-conjugated secondary antibodies at room temperature for 60 min. Blots were developed using ChemiLucent Detection System Kit (Millipore Chemicon International Inc., Temecula, CA, USA), and protein bands were visualized on X-ray film. Semiquantitation of the protein was performed using Image-Pro Plus 5.0 software (Media Cybernetic, Bethesda, MD, USA). 

### 2.13. Statistical Analysis

All parameters were expressed as mean ± SE. Statistical analysis was performed using one-way ANOVA followed by the Tukey test for multiple comparisons. Differences with *P* < 0.05 were considered to be significant.

## 3. Results

### 3.1. HQJZ Protects against the Colonic Mucosal Macroscopic and Histological Injuries by TNBS

**Figure 1:**
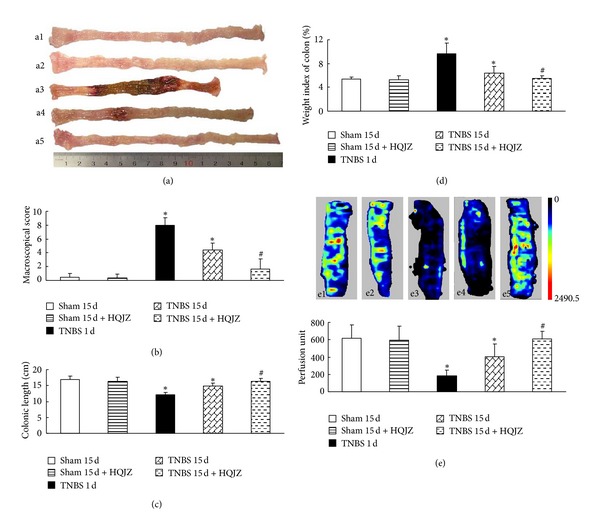
Macroscopic observation and colonic blood flow. (a) Representative images of colon, a1: Sham 15 d; a2: Sham 15 d + HQJZ; a3: TNBS 1 d; a4: TNBS 15 d; a5: and TNBS 15 d + HQJZ. (b) Macroscopic scores. (c) Colonic length. (d) Weight index of colon. (e) Representative images and quantitative analysis of colonic blood flow, e1: Sham 15 d; e2: Sham 15 d + HQJZ; e3: TNBS 1 d; e4: TNBS 15 d; and e5: TNBS 15 d + HQJZ. The magnitude of colonic blood flow is represented by different colors, with blue to red denoting low to high. Data were mean ± SEM (*n* = 8). *P < 0.05 versus Sham group; ^#^ *P* < 0.05 versus TNBS 15 d group.

**Figure 2:**
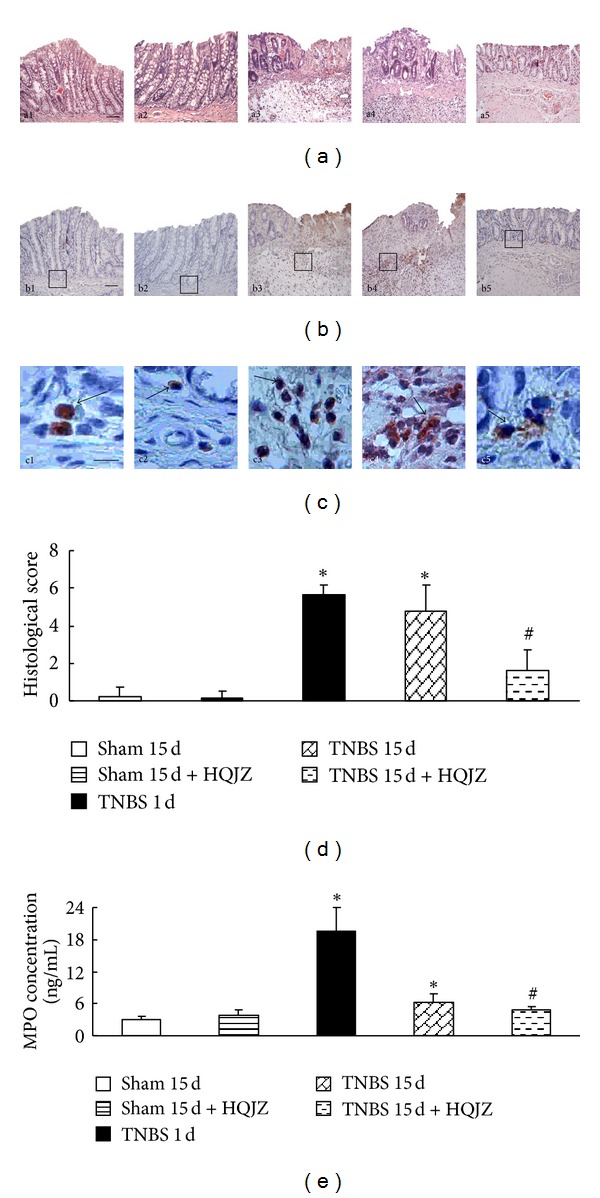
Representative histological images and scores and immunostaining and ELISA for MPO. (a) Representative histological images stained by HE, a1: Sham 15 d; a2: Sham 15 d + HQJZ; a3: TNBS 1 d; a4: TNBS 15 d; and a5: TNBS 15 d + HQJZ. Bar =100 *μ*m. (b) and (c) Immunostaining for MPO, b1 and c1: Sham 15 d; b2 and c2: Sham 15 d + HQJZ; b3 and c3: TNBS 1 d; b4 and c4: TNBS 15 d; and b5 and c5: TNBS 15 d + HQJZ. The arrowheads indicate MPO-positive cells. Bar (b1) = 100 *μ*m; Bar (c1) = 25 *μ*m. (d) Histological scores. (e) The concentration of MPO determined by ELISA. Data were mean ± SEM (*n* = 8). *P < 0.05 versus Sham group; ^#^ *P* < 0.05 versus TNBS 15 d group.

TNBS challenge for 1 d provoked apparent colonic mucosal injuries, including serious hyperemia, edema, and ulcers, some of which were covered with cruor or grimy sphacelus on the surface of colonic mucosa (Figure 1(a), a3). These injuries persisted till day 15 (Figure 1(a), a4) but attenuated by treatment with HQJZ (Figure 1(a), a5). Of notice, the colonic length in TNBS 1 d and TNBS 15 d groups was shorter than those in the 2 Sham groups, as well as shorter than that in TNBS 15 d + HQJZ group, indicating the protective role of HQJZ (Figures 1(a) and 1(c)). The colonic weight index and macroscopical injury scores were higher (Figures 1(b) and 1(d)) in TNBS 1 d and TNBS 15 d groups, compared to Sham groups, all of which were ameliorated significantly by HQJZ treatment. The representative microscopic images in different groups are shown in Figure 2(a). The histology in TNBS 1 d (a3) and TNBS 15 d (a4) groups exhibited pronounced alterations compared to Sham groups, including epithelial necrosis, epithalaxy, impaired mucosa involving submucosa with hyperemia and edema, and ulceration accompanied with numerous inflammatory cell infiltrations. Noticeably, all these alterations were alleviated in TNBS 15 d + HQJZ group (a5). Evaluation by histological scores confirmed this result, revealing a significant improvement of histology in TNBS 15 d + HQJZ group compared with TNBS-alone group (Figure 2(d)).

### 3.2. HQJZ Inhibits MPO Expression in Colonic Mucosa

MPO expression in colonic mucosa was assessed by ELISA while MPO-immunoreactive cells were evaluated by immunohistochemistry. MPO-positive cells with buffy particles were observed more frequently in the colonic stratum supra-vascular and submucosa in TNBS 1 d and TNBS 15 d groups compared to Sham group, whereas the number of MPO-immunoreactive cells decreased in TNBS 15 d + HQJZ group obviously (Figures 2(b) and 2(c)). Similarly, concentration of MPO tested by ELISA in the colonic tissue supernatant in TNBS 1 d and TNBS 15 d groups increased notably, which was significantly inhibited by HQJZ treatment (Figure 2(e)). These results indicated most of infiltrated cells as neutrophils.

### 3.3. HQJZ Improves Microcirculation of Colon

**Figure 3:**
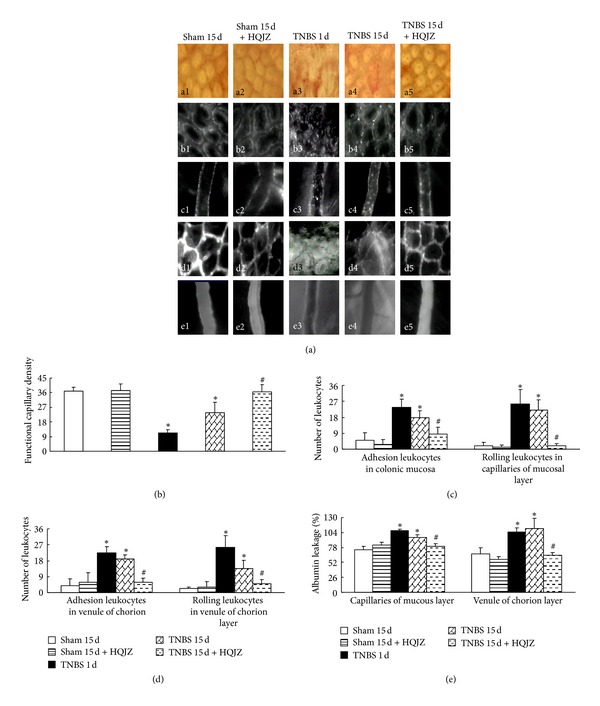
Colonic microcirculation. (a) Representative images of colonic microcirculation in different groups. a1–a5: Representative distribution of capillaries in colonic mucosa from different groups. b1–b5: Leukocyte adhesion to venules in mucous layer. c1–c5: Representative images of leukocyte adhesion to the wall of venules in chorion layer. d1–d5: Albumin leakage from venules in mucous layer. e1–e5: Representative images of albumin leakage from venules in chorion layer. Bar = 50 *μ*m. (b) The density of functional capillaries in different groups. (c) The number of rolling and adherent leukocytes in venules in mucous layer. (d) The number of rolling and adherent leukocytes in venules of chorion layer. (e) Statistic analysis of albumin leakage from venules in chorion and mucous layer. Data were mean ± SEM (*n* = 6). *P < 0.05 versus Sham group;  ^#^ *P* < 0.05 versus TNBS 15 d group.

Assessment by a laser Doppler perfusion imager (Figure 1(e)) showed a significant reduction in colonic blood flow in TNBS 1 d and TNBS 15 d groups compared to Sham group, which was reversed by HQJZ treatment. In parallel with this result, TNBS significantly reduced functional capillary density (*P* < 0.05 versus Sham), which was also ameliorated by HQJZ (Figures 3(a), a1–a5, and 3(b)).

Leukocyte rolling and adhesion were evaluated in capillaries of mucous layer and venules of chorion layer (Figures 3(a), b1–b5, c1–c5, and 3(c), and 3(d)). Clearly, few rolling and adherent leukocytes were observed in Sham and HQJZ-alone groups. In contrast, the number of rolling and adhered leukocytes increased remarkably in TNBS 1 d and 15 d groups. Treatment with HQJZ significantly attenuated TNBS-provoked leukocyte rolling and adhesion. Transvascular efflux of FITC-labeled albumin from capillaries of mucous layer and venules of chorion layer was detected in all groups (Figures 3(a), d1–d5, e1–e5, and 3(e)). The results demonstrated that albumin leakage in TNBS 1 d and TNBS 15 d groups remarkably increased, which was also attenuated significantly by treatment with HQJZ. 

### 3.4. HQJZ Inhibits CD11b Expression on Neutrophils, Decreases the Level of TNF-*α* and IL-6, and Increases the Level of IL-10 in Colonic Mucosa

**Figure 4:**
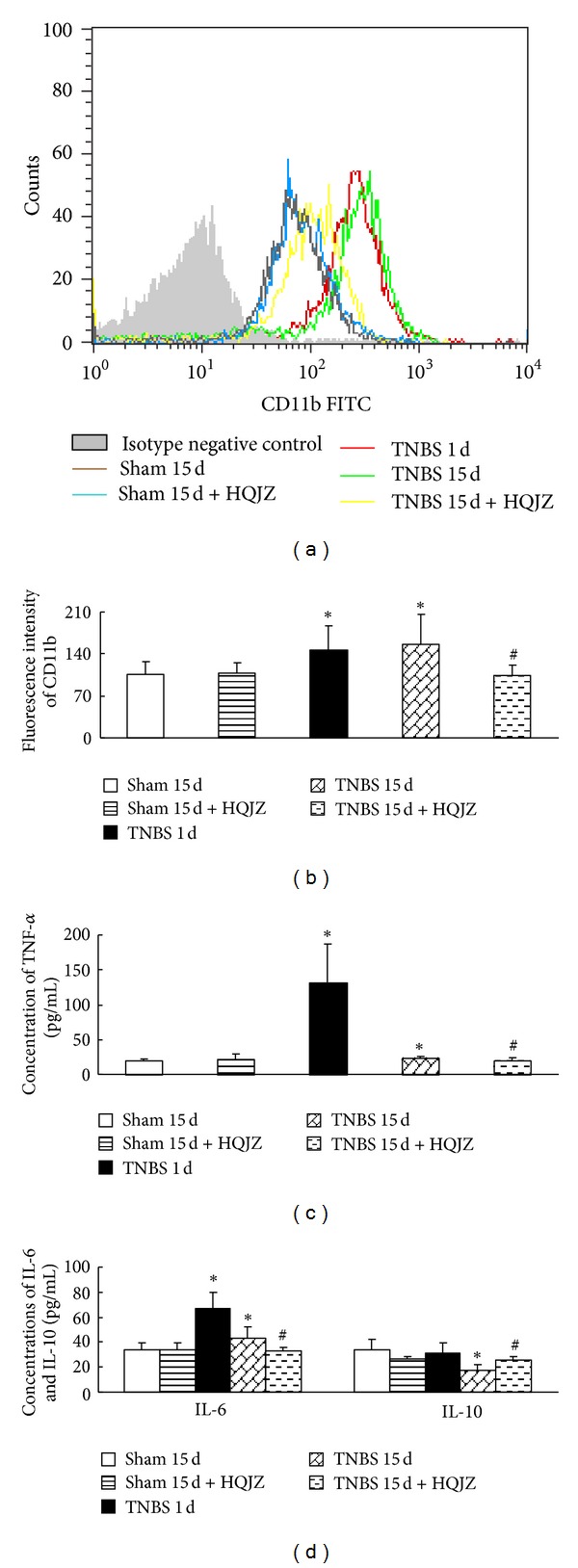
Expression of CD11b on neutrophils and the concentrations of cytokines in colonic mucosa. (a) Representative flow plots of CD11b on neutrophils in different groups. Histograms show the distribution of immunofluorescence labeling intensity of CD11b expression in each group. Ordinate indicates cell counts; Abscissa represents fluorescent intensity. (b) Fluorescence intensity of CD11b on neutrophils from different groups. (c) Concentration of TNF-*α* in colonic mucosa from different groups. (d) Concentrations of IL-6 and IL-10 in colonic mucosa from different groups. Data were mean ± SEM (*n* = 8). *P < 0.05 versus Sham group; ^#^ *P* < 0.05 versus TNBS 15 d group.

Assessment by flow cytometry revealed an enhanced expression of adhesion molecule CD11b on neutrophils in TNBS-induced rats. This enhancement was blunted significantly by HQJZ treatment (Figures 4(a) and 4(b)). 

The concentrations of the cytokines TNF-*α*, IL-6, and IL-10 in colonic tissues determined by cytometric bead array are presented in Figures 4(c) and 4(d). Compared with Sham groups, concentrations of TNF-*α* and IL-6 were elevated significantly in TNBS 1 d group and were reduced, but still statistically higher than those of Sham groups, in TNBS 15 d group. On the other hand, IL-10 was reduced in TNBS 15 d group, but not in TNBS 1 d group. TNBS-induced alteration in the concentration of cytokines observed in TNBS 15 d group was alleviated significantly by HQJZ treatment. 

### 3.5. HQJZ Attenuates the Degradation of Occludin in Colonic Mucosa

**Figure 5:**
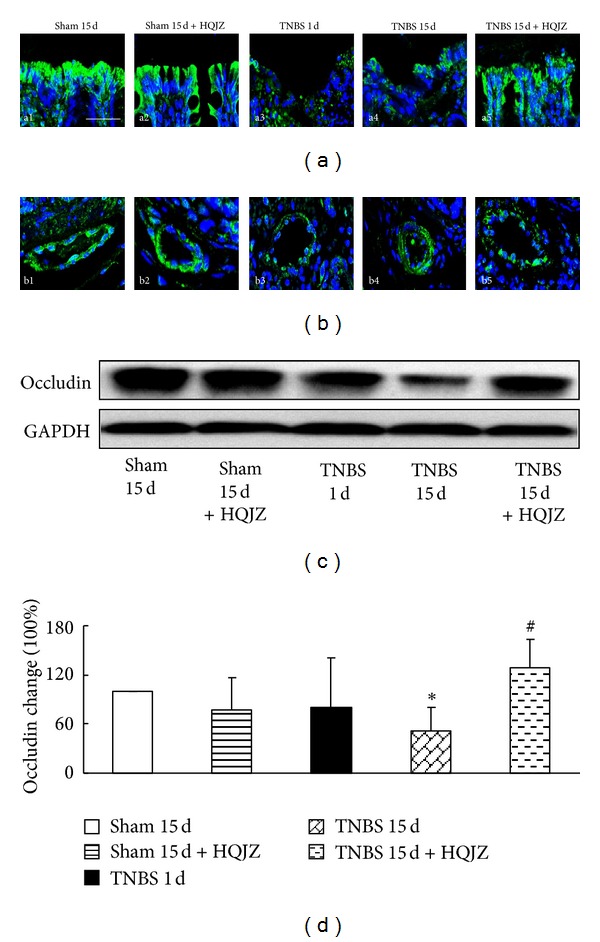
Representative immunofluorescence confocal images and Western blot analysis of occludin. Representative immunofluorescence confocal images of occludin in colonic mucosa (a) and venules from different groups (b). The green zone represents the distribution of occludin and the blue zone nuclei. Bar = 25 *μ*m. (c) Representative Western blot of occludin protein. (d) Quantitative analysis of occludin protein in colonic mucosa from different groups. Data were mean ± SEM (*n* = 5). *P < 0.05 versus Sham group; ^#^ *P* < 0.05 versus TNBS 15 d group.

Occludin was examined by confocal microscopy and Western blot. Confocal microscopy revealed a nearly continuous distribution of occludin on the surface of colonic epithelium and the junctions of colonic epithelial cells, as well as on the endothelium of microvessels in Sham groups. The distributions of occludin became discontinuous in TNBS 1 d and TNBS 15 d groups. HQJZ treatment for 14 days apparently restored the alteration in occludin distribution caused by TNBS (Figures 5(a) and 5(b)). The results were confirmed by Western blot analysis (Figures 5(c) and 5(d)), showing a noticeable decrease in occludin expression in colonic mucosa from rats in TNBS 15 d group, while HQJZ treatment significantly relieved the decrease of occludin in the colonic mucosa 15 days after TNBS challenge. 

### 3.6. HQJZ Regulates the Colonic Energy Status and Distribution of F-Actin

**Figure 6:**
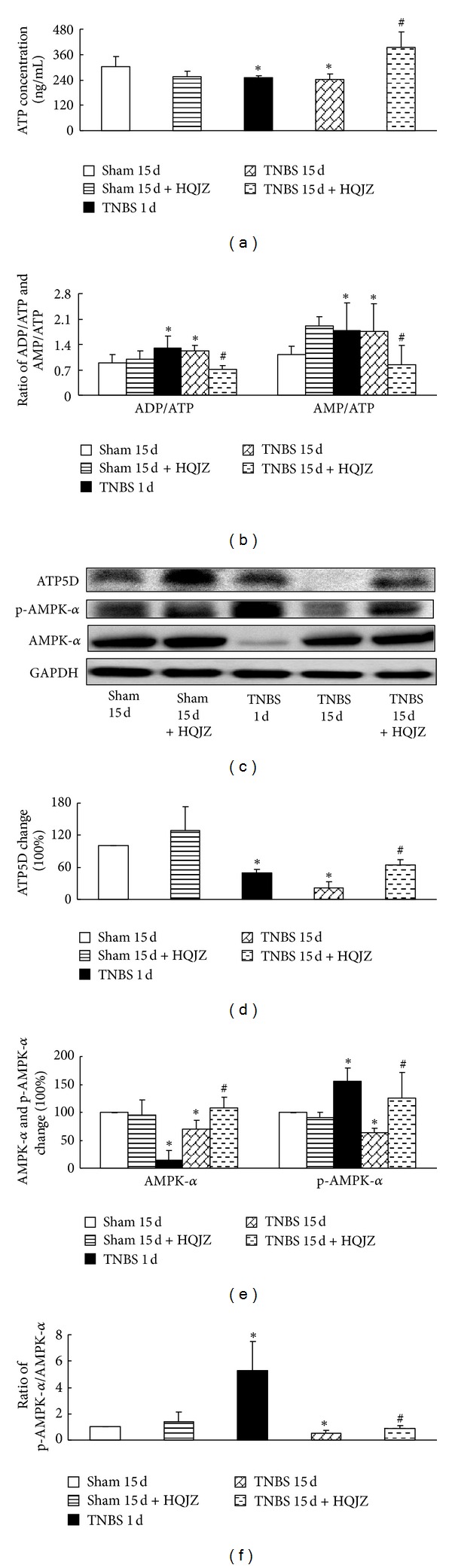
Energy status of colonic mucosa and Western blot analysis of ATP5D, AMPK-*α*, and p-AMPK-*α*. (a) The concentration of ATP in colonic mucosa (*n* = 8). (b) The ratios of ADP/ATP and AMP/ATP (*n* = 8). (c) Representative Western blot of ATP5D, AMPK-*α*, p-AMPK-*α* and GAPDH (*n* = 5). (d) Quantitative analysis of ATP5D protein (*n* = 5). (e) Quantitative analysis of AMPK-*α* and p-AMPK-*α* proteins. (f) The ratio of p-AMPK-*α*/AMPK-*α* (*n* = 5). Data were mean ± SEM. *P < 0.05 versus Sham group; ^#^ *P* < 0.05 versus TNBS 15 d group.

The content of ATP, ADP, and AMP was analyzed by ELISA (Figures 6(a) and 6(b)). The concentration of ATP in colonic tissue supernatant decreased notably after TNBS challenge for 1 day and 15 days. Correspondingly, the ratio of ADP/ATP and AMP/ATP was upregulated apparently. These TNBS-induced alterations in concentration of ATP and ratio of ADP/ATP and AMP/ATP were restored significantly by treatment with HQJZ (Figures 6(a) and 6(b)).

AMPK-*α* expression and phosphorylation and ATP5D expression were analyzed by Western blot (Figures 6(c), 6(d), 6(e), and 6(f)), revealing a considerable decrease in the expression of ATP5D protein in TNBS-challenged rats without HQJZ treatment. On the other hand, TNBS-evoked alteration in AMPK-*α* was more perplexing in that AMPK-*α* was decreased prominently in TNBS 1 d group, recovered somewhat but still statistically lower than Sham groups in TNBS 15 d group. While phospho-AMPK-*α* increased obviously in TNBS 1 d group, it decreased in TNBS 15 d group, as compared to Sham groups. Nonetheless, HQJZ treatment for 14 days significantly attenuated all of the alterations in ATP5D protein, AMPK-*α*, and phospho-AMPK-*α* caused by TNBS challenge.

**Figure 7:**
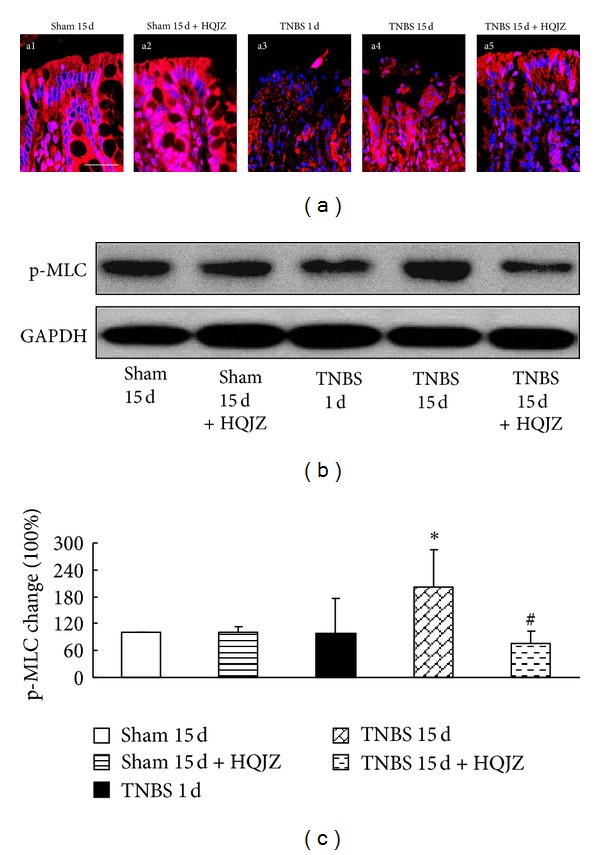
Distribution of F-actin in colonic mucosa and Western blot analysis of p-MLC. (a) Distribution of F-actin in colonic mucosa. The red zone represents the distribution of F-actin, and the blue zone represents nuclei. Bar = 25 *μ*m. (b) Representative Western blots of p-MLC and GAPDH. (c) Quantitative analysis of p-MLC proteins. Data were mean ± SEM (*n* = 5). *P < 0.05 versus Sham group; ^#^ *P* < 0.05 versus TNBS 15 d group.

We investigated the expression of F-actin by confocal microscopy, and MLC phosphorylation by Western blot. Confocal microscopy (Figure 7(a)) revealed that F-actin expression decreased in the colonic epithelium from TNBS-challenged rats compared to that from Sham group. HQJZ treatment apparently attenuated F-actin expression (Figure 7(a), a5). Moreover, MLC phosphorylation enhanced pronouncedly in response to 15 days of TNBS challenge, which was restored significantly by HQJZ treatment (Figures 7(b) and 7(c)). 

### 3.7. HQJZ Regulates the Balance of RhoA/Rac in Colonic Mucosa

**Figure 8:**
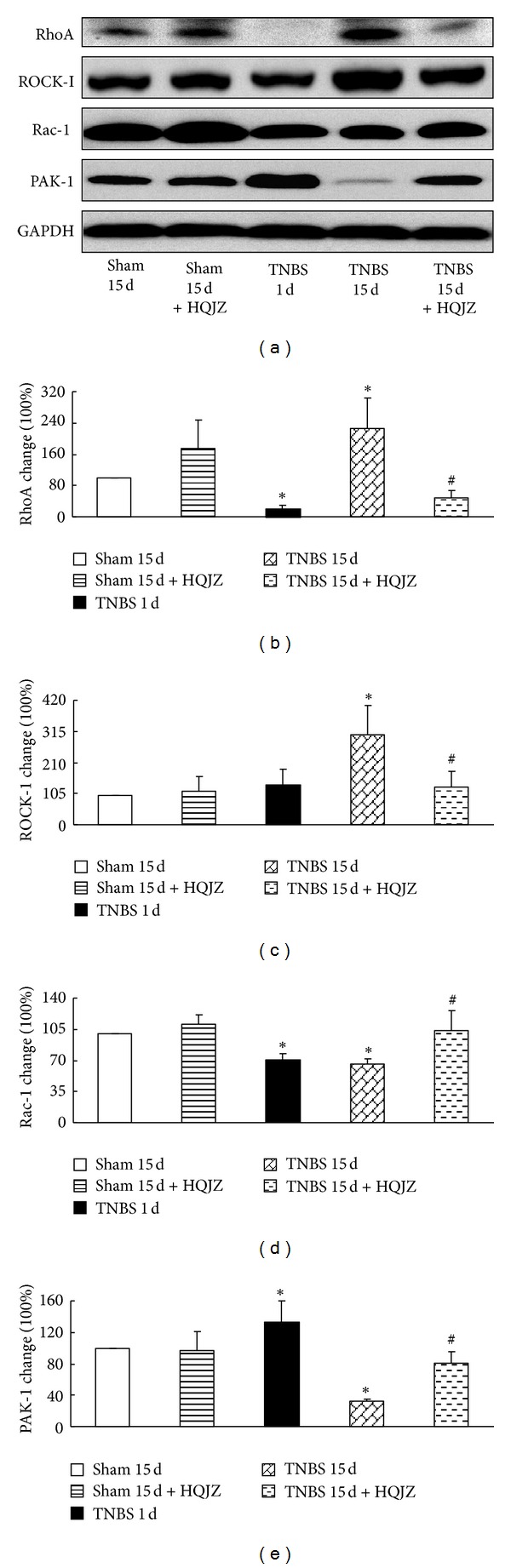
Western blot analysis of RhoA, ROCK-I, Rac-1, and PAK-1. (a) Representative Western blots of RhoA, ROCK-I, Rac-1, PAK-1, and GAPDH. (b) Quantitative analysis of RhoA protein. (c) Quantitative analysis of ROCK-I protein. (d) Quantitative analysis of Rac-1 protein. (e) Quantitative analysis of PAK-1 protein. Data were mean ± SEM (*n* = 5). *P < 0.05 versus Sham group; ^#^ *P* < 0.05 versus TNBS 15 d group.

The expressions of RhoA, ROCK-I, Rac-1, and PAK-1 proteins were assessed by Western blot. Compared to Sham groups, TNBS challenge for 15 days evoked a significant increase in the expressions of RhoA (Figures 8(a) and 8(b)) and ROCK-I (Figures 8(a) and 8(c)), while it showed a decrease in Rac-1 (Figures 8(a) and 8(b)) and PAK-1 (Figures 8(a) and 8(e)). HQJZ restrained all of the TNBS-evoked alterations significantly.

## 4. Discussion

TNBS-induced murine colitis is an extensively used animal model for human IBD. In the present study, TNBS administration successfully evoked colonic inflammation in rats, as evidenced by both macro- and microscopic manifestations, as well as by the colonic microcirculatory disturbance and alterations in inflammatory cytokine production in colonic tissue. Importantly, all of the TNBS-evoked insults were attenuated by HQJZ treatment, highlighting its therapeutic effects on TNBS-induced colonic mucosal injury. 

As a famous Chinese medicine formula, although HQJZ has been used in China almost for two thousand years, the study on the mechanisms responsible for its role in inflammatory diseases remains limited, and most of works were focusing on its major component *Astragalus*. Consistent with the results from studies on *Astragalus*, the present study revealed HQJZ to be able to downregulate the production of proinflammatory cytokines and upregulate anti-inflammatory cytokines. Furthermore, the current study demonstrated that HQJZ inhibited TNBS-induced CD11b expression on leukocytes and leukocyte adhesion to venular wall. The mechanism responsible for the anti-inflammatory potential of HQJZ is not clear at present. Nonetheless, the antioxidant ability of its compositions may contribute, at least in part, to the beneficial role of HQJZ in this regard [25–27].

In addition, HQJZ was found to attenuate the albumin leakage and leukocyte emigration from venules in the presence of TNBS, indicating its role in protection against vascular hyperpermeability. The significance of this finding resides in that, in colonic inflammation, hyperpermeability occurs not only in vascular endothelium but also in mucosal epithelium. Patients with colonic inflammatory disease typically present with relapsing diarrhea, which has been attributed to increased paracellular permeability in the colonic epithelium [44]. Thus, a management able to restore the increased colonic epithelium permeability is of great significance for relieving the symptom, particularly the malnutrition that patients suffer from. The permeability of the endothelial and epithelial barrier is controlled by intercellular junctions. The present study assessed the effect of HQJZ on the occludin, a protein that stabilizes tight junction through interaction with ZO-1 and actin cytoskeleton [45]. As expected, the result showed that HQJZ affected barrier permeability via mechanism(s) involving modulation of both expression and distribution of occludin.

 We next explored the signaling pathway for the role of HQJZ in regulating barrier function. In regulation of barrier of both endothelium and epithelium, the Rho family, RhoA, Rac, and Cdc42 have been recognized as a major player [16–21]. The results from the present study showed that TNBS challenge evoked an increase in RhoA and ROCK-1, as well as in p-MLC, while it showed a decrease in Rac and PAK-1. Interestingly, all of these alterations were attenuated by treatment with HQJZ. These results highlight an implication of Rho family in the role of HQJZ in maintaining endothelial and epithelial barrier function.

Complete remission of colonic inflammatory diseases requires both the relief of inflammation and the repair of damaged epithelium. The repair of damaged colonic mucosa initiates with cell restitution, characterized by cell spreading and migration into the wound to restore epithelial continuity. We observed that HQJZ promoted the repair of damaged epithelium as evidenced by macro- and microscopic findings, implying a potential of HQJZ to accelerate cell restitution. It is likely that HQJZ exerts this effect via modulation of Rho family, since cell restitution is a process of cellular locomotion, which has been shown to be driven by Rho family [20].

It appears that the signaling pathways mediating the role of HQJZ in attenuating endothelial and epithelial barrier function and in promoting cell restitution both converge on Rho family. The question then arises: How does HQJZ regulate Rho family? In view of the critical importance of energy metabolism in regulation of Rho family, we assumed that HQJZ may regulate Rho family by interference in energy metabolism.

We tested this assumption first by assessment of ATP, ADP, and AMP content in different condition. The result revealed a significant increase in ATP content in TNBS-injured rats when subjected to HQJZ treatment, suggesting the capacity of HQJZ to increase the energy supply, consistent with the result from others [31]. Energy metabolism is a process involving multiple reactions coordinated by numerous proteins, among which ATP synthase plays a central role, while AMPK acts as an energy sensor to monitor the energy status of the cell [46]. In the present study we assessed ATP5D, a critical subunit of ATP synthase [31], and revealed a significant decrease in ATP5D in colonic mucosa tissue after TNBS challenge, which accounts for the insufficient ATP supply observed then. The examination of AMPK showed an increase in activated AMPK in the early phase in injuring process by TNBS (1 day), reflecting an attempt of the cell to compensate the lack of energy, and it showed a decrease in the late phase (15 days), implying a failure of this attempt which led to delayed tissue injury repair. Interestingly, treatment with HQJZ resulted in an increased expression of ATP5D in colonic tissue damaged by TNBS, while it had little, though statistically significant, influence on the activated AMPK content compared to TNBS-alone group. This result implied that HQJZ modulated energy metabolism mostly by enhancing the expression of ATP5D, and, to a less extent, by activating AMPK. The mechanism for HQJZ to affect the expression of ATP5D and activation of AMPK needs to be elucidated by further study.

## 5. Conclusions

In summary, using TNBS-induced rat colonic mucosal injury as a model, the present study verified the favorable role of HQJZ in IBD, which manifested as attenuation of microcirculatory disturbance, relief of inflammation and colonic epithelium barrier function, and improvement of energy supply. HQJZ exerted its effects most likely by acting as an antioxidant and an energy metabolism modulator, suggesting it as a multitargeting strategy. Nonetheless, the detailed mechanism remains to be identified by further study.

## Figures and Tables

**Table 1:** Characterization of the herbs included in HQJZ Pellet.

Herbs	Percentage content (%)	Identified compounds	Effects	References
Radix astragali mongolici (Huang Qi)	12.50	Astragaloside	Inhibiting NF-*κ*B signaling and triggering T cell activation, antioxidative	[22, 23, 26]
Cortex cinnamomi cassiae (Rou Gui)	8.33	Cinnamaldehyde	Antisepsis	[24]
Radix paeoniae alba (Bai Shao)	12.50	Paeoniflorin	Antioxidant	[25]
*Fructus jujubae* (Da Zao)	8.33	Oleanolic acid	Antioxidative, antiglycative, and antiapoptotic effects	[27]
Rhizoma zingiberis recens (Sheng Jiang)	8.33	Volatile oil	Gastroprotective effects	[28]
Glycyrrhiza uralensis fisch (Gan Cao)	8.33	Glycyrrhizic acid	Against endothelial dysfunction	[29]
Saccharum granorum (Yi Tang)	41.66	Maltose and dextrin	Increasing free-energy (ATP) conservation	[32]

**Table 2:** The number of animals in different experimental groups for various parameters.

	Sham 15 d	Sham 15 d + HQJZ	TNBS 1 d	TNBS 15 d	TNBS 15 d + HQJZ	Total
Colonic microcirculation	6	6	6	6	6	30
Colonic blood flow, macroscopical and microscopical evaluation, expression of CD11b and, cytokines	8	8	8	8	8	40
Immunohistochemistry and immnofluorescence staining	(3)	(3)	(3)	(3)	(3)	
Western blot analysis	(5)	(5)	(5)	(5)	(5)	

Total	14	14	14	14	14	70

The animals were separated as two batches in the present study. Only one batch of animals (30 rats) was used to observe colonic microcirculation. And the other parameters were analyzed using the second batch of animals (40 rats). The same animals were used for detection of colonic blood flow, macroscopical and microscopical evaluation, and expression of CD11b and cytokines. Brackets: the tissues for immunohistochemistry, immnofluorescence staining, and Western blotting analysis were removed from the second batch of animals (40 rats). Sham 15 d: the rats received physiological saline by enema and 24 h thereafter physiological saline by: gavage for 14 days; Sham 15 d + HQJZ: the rats received physiological saline by enema and 24 h thereafter HQJZ at 2 g/kg by gavage for 14 days; TNBS 1 d, the rats received TNBS by enema, and were sacrificed after 24 h; TNBS 15 d: the rats received TNBS by enema and 24 h thereafter: physiological saline by gavage for 14 days; TNBS 15 d + HQJZ: the rats received TNBS by enema and 24 h thereafter: HQJZ at 2 g/kg everyday by gavage for 14 days.
